# Production of recombinant VP1-derived virus-like particles from novel human polyomaviruses in yeast

**DOI:** 10.1186/s12896-015-0187-z

**Published:** 2015-08-04

**Authors:** Milda Norkiene, Jomante Stonyte, Danguole Ziogiene, Egle Mazeike, Kestutis Sasnauskas, Alma Gedvilaite

**Affiliations:** Institute of Biotechnology, Vilnius University, Graiciuno 8, LT-02241 Vilnius, Lithuania

**Keywords:** Human polyomavirus, VP1, Virus-like particles, Yeast, Hemagglutination assay

## Abstract

**Background:**

Eleven new human polyomaviruses (HPyVs) have been identified in the last decade. Serological studies show that these novel HPyVs sub-clinically infect humans at an early age. The routes of infection, entry pathways, and cell tropism of new HPyVs remain unknown. VP1 proteins of polyomaviruses can assembly into virus-like particles (VLPs). As cell culturing systems for HPyV are currently not available, VP1-derived VLPs may be useful tools in basic research and biotechnological applications.

**Results:**

Recombinant VP1-derived VLPs from 11 newly identified HPyVs were efficiently expressed in yeast. VP1 proteins derived from Merkel cell polyomavirus (MCPyV), trichodysplasia spinulosa-associated polyomavirus (TSPyV), and New Jersey polyomavirus (NJPyV) self-assembled into homogeneous similarly-sized VLPs. Karolinska Institutet polyomavirus (KIPyV), HPyV7, HPyV9, HPyV10, and St. Louis polyomavirus (STLPyV) VP1 proteins formed VLPs that varied in size with diameters ranging from 20 to 60 nm. Smaller-sized VLPs (25–35 nm in diameter) predominated in preparations from Washington University polyomavirus (WUPyV) and HPyV6. Attempts to express recombinant HPyV12 VP1-derived VLPs in yeast indicate that translation of VP1 might start at the second of two potential translation initiation sites in the VP1-encoding open reading frame (ORF). This translation resulted in a 364-amino acid-long VP1 protein, which efficiently self-assembled into typical PyV VLPs. MCPyV-, KIPyV-, TSPyV-, HPyV9-, HPyV10-, and HPyV12-derived VLPs showed hemagglutination (HA) assay activity in guinea pig erythrocytes, whereas WUPyV-, HPyV6-, HPyV7-, STLPyV- and NJPyV-derived VP1 VLPs did not.

**Conclusions:**

The yeast expression system was successfully utilized for high-throughput production of recombinant VP1-derived VLPs from 11 newly identified HPyVs. HPyV12 VP1-derived VLPs were generated from the second of two potential translation initiation sites in the VP1-encoding ORF. Recombinant VLPs produced in yeast originated from different HPyVs demonstrated distinct HA activities and may be useful in virus diagnostics, capsid structure studies, or investigation of entry pathways and cell tropism of HPyVs until cell culture systems for new HPyVs are developed.

## Background

Polyomaviruses (PyVs) are small, non-enveloped viruses with a circular, double-stranded genome comprised of approximately 5000 base pairs surrounded by an icosahedral capsid [1]. PyVs have been found to infect both mammalian (*Orthopolyomavirus* and *Wukipolyomavirus*) and avian (*Avipolyomavirus*) species [2, 3]. Mammalian PyV infections are usually asymptomatic, although PyVs were originally identified and named for their ability to cause multiple types of tumors [4]. BK polyomavirus (BKPyV) and JC polyomavirus (JCPyV) were the first two human PyVs (HPyVs) isolated 40 years ago [5, 6]. As a result of improved molecular diagnostic techniques, combined with strategies in sample preparation to enrich viral DNA content through reduction of host genomic DNA, 11 new HPyVs have been identified in the last decade. Of these novel HPyVs, Karolinska Institutet polyomavirus (KIPyV), Washington University polyomavirus (WUPyV), and Merkel cell polyomavirus (MCPyV) were among the first to be discovered [7–9]. Later, HPyV6, HPyV7, trichodysplasia spinulosa-associated polyomavirus (TSPyV), and HPyV9 were identified [10–12]. By 2014, three more HPyVs (HPyV10, St. Louis polyomavirus (STLPyV), and HPyV12) were discovered [13–15]. Two other PyVs detected in human stool samples, Malawi polyomavirus (MWPyV) and Mexico polyomavirus (MXPyV), may represent strain variants of HPyV10 because their genomes are 95–99 % identical [16, 17]. The latest virus, New Jersey polyomavirus (NJPyV), was discovered in endothelial cells, sites of myositis, and sites of cutaneous necrosis in a pancreatic transplant recipient [18].

Discoveries of new HPyVs have revived interest in this expanding family of human pathogens. There are several reasons why HPyVs may be important in human pathology. First, according to serological studies, HPyVs sub-clinically infect humans at an early age, with rates ranging from 35 to 90 % [19, 20]. Secondly, while HPyV infection is generally asymptomatic, it can cause serious illness in immunocompromised individuals [19]. Accordingly, the involvement of some HPyVs in the pathology of various diseases has been reported. Infection by and replication of BKPyV in kidney epithelial cells can lead to PyV-associated nephropathy [21, 22]. Progressive multifocal leukoencephalopathy is caused by uncontrolled JCPyV replication in oligodendrocytes of the brain in immunosuppressed patients, including patients with advanced acquired immune deficiency syndrome (AIDS) [22, 23]. Trichodysplasia spinulosa, a rare skin dysplasia, occurs in immunocompromised individuals as a result of TSPyV replication in the inner root sheath cells of hair follicles [11]. HPyV7 has also been associated with novel pathogenicity: lower back rash in immunosuppressed patients [24]. Thirdly, several PyVs may be associated with human cancer. MCPyV has gained the most attention due to its link with a rare and aggressive malignant human skin cancer, Merkel cell carcinoma, in the elderly and chronically immunosuppressed individuals [19]. It has been shown that hamster polyomavirus (HaPyV) is responsible for the high incidence of lymphomas in a colony of genetic audiogenic seizure hamsters (GASH:Sal) [25]. Moreover, proteins encoded by some PyVs have transforming ability in cell culture and tumorigenic activity in animals [26]. The connection with disease of other new HPyVs has yet to be determined.

The late gene region of mammalian PyVs encode three structural proteins, VP1, VP2, and VP3, which together make up the viral capsid; the capsid of avian PyVs may additionally contain a unique VP4 protein [1, 27]. However, capsid-like structures can assemble of the major capsid protein VP1 alone [28–30]. This unique property of VP1 makes it an ideal protein for generating virus-like particles (VLPs). VLPs resemble the native virions they are derived from in structure, immunogenicity, and tropism, but they do not contain any viral genetic material. VLPs are particularly valuable in various research applications because many PyVs are not easily cultured, which may be related to the finding that PyVs infect only a very limited number of species and cell types in nature [1]. PyV VP1-derived VLPs play an important role in the study of mechanisms underlying protein self-assembly, attachment to host cell receptors, virus entry, immune response, intracellular trafficking, and in the epidemiologic study and diagnosis of PyV infection [1, 31]. VP1-derived VLPs may also have potential use in vaccine development, gene delivery in gene therapy, and other biomedical purposes [31, 32]. Sequence alignment of VP1 from different HPyVs shows high amino acid (aa) sequence identity between some HPyVs, resulting in occasional antibody cross-reactivity [20]. Cross-reactivity has been observed between HPyV6 and HPyV7 (69 % identity) or BKPyV and JCPyV (78 % identity), but not between MCPyV, TSPyV, HPyV9, or MWPyV [20, 33, 34].

We have previously demonstrated that Simian virus 40 (SV40)-, HaPyV-, BKPyV-, JCPyV-, and murine polyomavirus (MPyV)-derived VP1 VLPs can be efficiently produced in the yeast (*Saccharomyces cerevisiae*) strain, AH22-214, using the pFX7 expression plasmid [29, 30]. In this study, we showed that the applied yeast system is also suitable for efficient expression and self-assembly of VP1-derived VLPs from 11 novel HPyVs: MCPyV, KIPyV, WUPyV, HPyV6, HPyV7, TSPyV, HPyV9, MWPyV, STLPyV, HPyV12, and NJPyV. Yeast-expressed recombinant VLPs were subjected to a hemagglutination (HA) assay, which our findings suggest might be useful in developing new HPyV diagnostic tools and in other applications.

## Results

### Production of VP1-derived VLPs from MCPyV, KIPyV, WUPyV, HPyV6, HPyV7, TSPyV, HPyV9, HPyV10, STLPyV and NJPyV in yeast

For the production of KIPyV-, WUPyV-, HPyV6-, HPyV7-, TSPyV-, HPyV9-, STLPyV-, and NJPyV-derived VLPs in yeast, codons in the VP1-encoding open reading frames (ORFs) from corresponding HPyV genomes were optimized for expression in *S. cerevisiae.* A few deviations from strict, high-frequency codon usage were allowed for the insertion or removal of restriction endonuclease recognition sites. HPyV10 VP1 gene was synthesized according to the native virus gene sequence without codon optimization. MCPyV VP1 gene was amplified by polymerase-chain reaction (PCR) from the MCPyV virus genome cloned from a healthy volunteer skin swab. All 10 VP1-encoding genes were inserted in the same site in the expression vector, pFX7 [29], under the control of a galactose-inducible promoter.

**Fig. 1:**
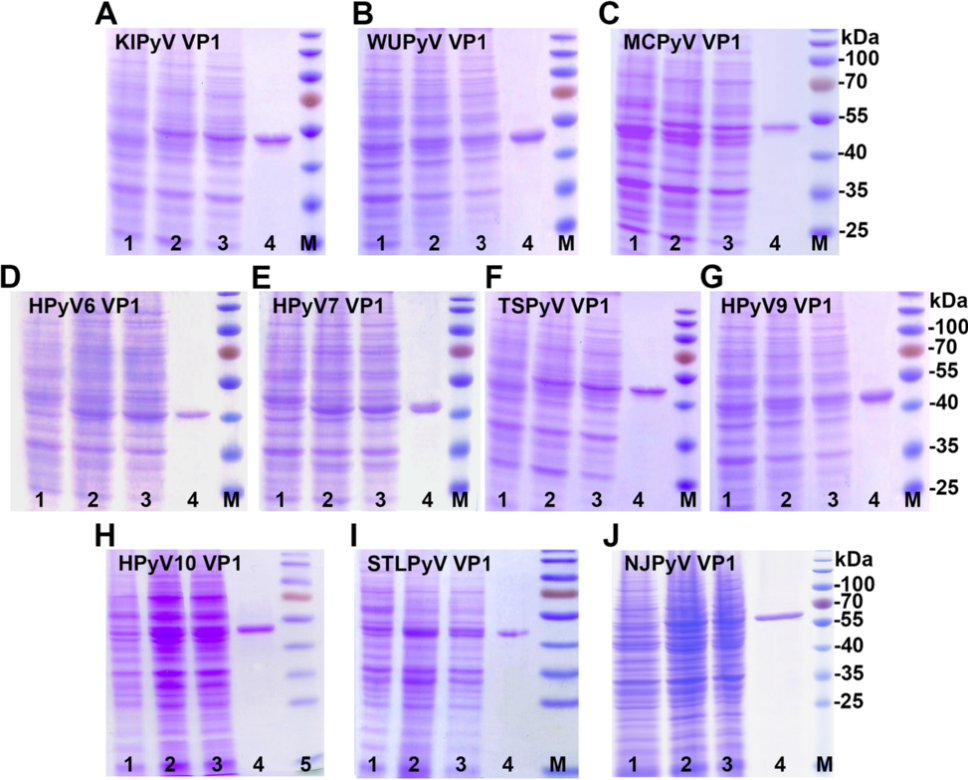
Analysis of the production of novel HPyV-derived VP1 proteins in yeast using Coomassie blue-stained SDS-PAGE. In lanes: 1- in all gels, negative control sample of whole crude lysate of yeast transformed with pFX7 plasmid; 2 - whole lysate of yeast transformed with pFX7-KIPyV-VP1 (**a**), pFX7-WUPyV-VP1 (**b**), pFX7-MCPyV-VP1 (**c**), pFX7-HPyV6-VP1 (**d**), pFX7-HPyV7-VP1 (**e**), pFX7-TSPyV-VP1 (**f**), pFX7-HPyV9-VP1 (**g**), pFX7-HPyV10-VP1 (**h**), pFX7-STLPyV-VP1 (**i**), or pFX7-NJPyV-VP1 (**j**) plasmid; 3 - the soluble fraction recovered after centrifugation of whole lysate of yeast transformed with pFX7-KIPyV-VP1 (**a**), pFX7-WUPyV-VP1 (**b**), pFX7-MCPyV-VP1 (**c**), pFX7-HPyV6-VP1 (**d**), pFX7-HPyV7-VP1 (**e**), pFX7-TSPyV-VP1 (**f**), pFX7-HPyV9-VP1 (**g**), pFX7-HPyV10-VP1 (**h**), pFX7-STLPyV-VP1 (**i**), or pFX7-NJPyV-VP1 (**j**) plasmid; 4 - purified KIPyV VP1 (**a**), WUPyV VP1 (**b**), MCPyV VP1 (**c**), HPyV6 VP1 (**d**), HPyV7 VP1 (**e**), TSPyV VP1 (**f**), HPyV9 VP1 (**g**), HPyV10 VP1 (**h**), STLPyV VP1 (**i**), or NJPyV VP1 (**j**) protein. In all gels, pre-stained protein weight marker (Thermo Fisher Scientific Baltics) was loaded into lane M

To determine whether recombinant VP1 protein was expressed in yeast transformed with pFX7 carrying HPyV VP1-encoding ORFs, yeast lysates were analyzed using sodium dodecyl sulfate-polyacrylamide gels (SDS-PAGE) (Fig. 1a–j, line 2). The expression of all 10 constructs was detected in yeast lysates, and the expression level of all VP1 proteins derived from different HPyVs did not vary significantly. All 10 recombinant VP1 proteins stayed in the soluble fraction after the centrifugation of yeast lysates (Fig. 1a–j, line 3). The solubility of VP1 allowed for the efficient purification by sucrose and CsCl gradient centrifugation (Fig. 1j, line 4). The yield of purified VP1-derived VLPs ranged from 0.44 to 1.05 mg per 1 g of wet yeast biomass (Table 1). The lower yields of purified VP1 from HPyV6, HPyV7, HPyV10, and NJPyV may have been the consequence of minor differences in expression level and solubility. On the other hand, it is known that codon usage might influence the efficiency of heterologous gene expression [35, 36]. Thus, the lower yield of HPyV10-derived VP1 could be a result of the HPyV10 VP1-encoding gene sequence, as it was not optimized for yeast expression. During purification, KIPyV-, HPyV6-, and HPyV7-derived VP1 tended to degrade. Therefore, additional bands in the SDS-PAGE gel were observed (Fig. 1a, d, e, line 4). Highly-soluble TSPyV- and HPyV9-derived VP1 were purified with yields as high as 1 mg per 1 g of wet yeast biomass (Table 1).

**Table 1:** Characterization of the yeast-expressed novel HPyV-derived VP1 proteins

Viral origin of VP1	VP1 gene sequence	Length of VP1, aa	Predicted molecular weight, kDa	Formation of VLPs^a^	Yield of purified VP1 VLPs from 1 g of wet yeast biomass, mg^b^	HA of guinea pig erythrocytes
KIPyV	Optimized	378	41.59	+	0.62 ± 0.05	+
WUPyV	Optimized	369	39.87	+	0.61 ± 0.03	-
MCPyV	Native	423	46.56	+	0,81 ± 0.04	+
HPyV6	Optimized	387	41.84	+	0.55 ± 0.04	-
HPyV7	Optimized	380	40.98	+	0.58 ± 0.03	-
TSPyV	Optimized	375	41.52/	+	1.05 ± 0.04	+
HPyV9	Optimized	371	40,32	+	1.01 ± 0.03	+
HPyV10	Native	403	43.57	+	0.44 ± 0.04	+
STLPyV	Optimized	401	43.54	+	0.62 ± 0.04	-
HPyV12	Optimized	380	42.16	+/−	0.05 ± 0.03	+
HPyV12	Native	364	40.39	+	0.42 ± 0.04	+
HPyV12	Optimized	364	40.39	+	1.02 ± 0.06	+
NJPyV	Optimized	489	53.78	+	0.45 ± 0.03	-

^a^The formation of VLPs was confirmed by negative stain electron microscopy

^b^The values for the yield of VLPs are the average at least from three independent experiments

**Fig. 2:**
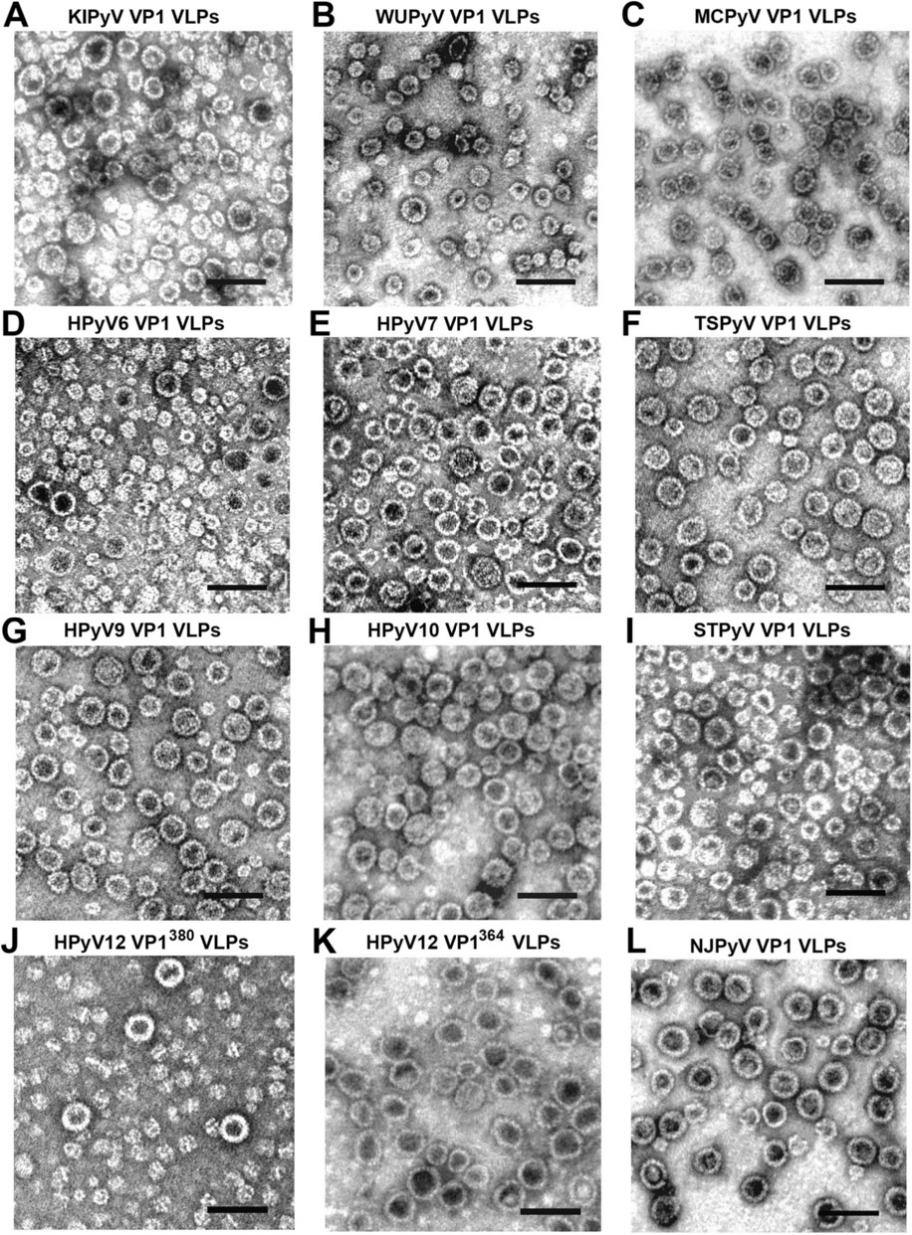
Detection of purified HPyV-derived VP1 VLPs using negative staining electron microscopy. Electron micrographs of: (**a**) - KIPyV VP1 VLPs, (**b**) - WUPyV VP1 VLPs, (**c**) - MCPyV VP1 VLPs, (**d**) - HPyV6 VP1 VLPs, (**e**) - HPyV7 VP1 VLPs, (**f**) - TSPyV VP1 VLPs, (**g**) - HPyV9 VP1 VLPs, (**h**) - HPyV10 VP1 VLPs, (**i**) - STLPyV VP1 VLPs, (**j**) - HPyV12 VP1^380^ VLPs, (**k**) - HPyV12 VP1^364^ VLPs, and (**l**) - NJPyV VP1 VLPs produced in yeast. Bars, 100 nm

The ability of VP1 proteins to self-assemble into VLPs was examined using negative staining electron microscopy. All 10 purified VP1 proteins formed VLPs (Fig. 2). MCPyV-, TSPyV-, and NJPyV-derived VLPs were found to be the most similar in size (Fig. 2c, f) with diameters of 45–50 nm (Fig. 2c, f, l). The particles formed by seven other VP1 proteins were more variable in size with diameters ranging from 25 to 55 nm. Although the majority of VLPs in preparations from KIPyV, HPyV7, HPyV9, HPyV10, and STLPyV were 40–50 nm in diameter (Fig. 2a, e, g, h, i), fractions of smaller VLPs, 25–35 nm in diameter, predominated in samples from WUPyV and HPyV6 (Fig. 2b, d).

### Production of HPyV12 VP1-derived VLPs in yeast

**Fig. 3:**
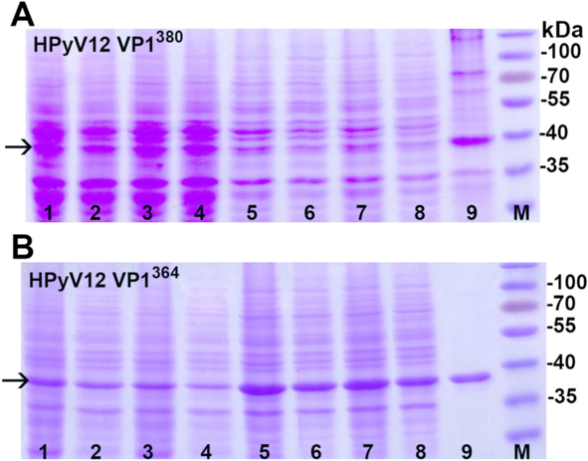
Analysis of HPyV12-derived VP1 VLP production in yeast. **a** - Analysis of HPyV12 VP1^380^ VLP production in yeast using Coomassie blue-stained SDS-PAGE. Production of HPyV12 VP1^380^ protein was analyzed in two yeast clones transformed with pFX7-HPyV12-VP1n^380^ plasmid (lines 1–4) and two clones transformed with pFX7-HPyV12-VP1s^380^ plasmid (lines 5–8). In lanes: 1, 3 - whole lysate of both yeast clones transformed with pFX7-HPyV12-VP1n^380^ plasmid; 2, 4 - the soluble fraction recovered after centrifugation of whole lysate of both yeast clones transformed with pFX7-HPyV12-VP1n^380^ plasmid; 5, 7 - whole lysate of both yeast clones transformed with pFX7-HPyV12-VP1s^380^ plasmid; 6, 8 - the soluble fraction recovered after the centrifugation of whole lysate of both yeast clones transformed with pFX7-HPyV12-VP1s^380^ plasmid; 9 - purified HPyV12-VP1^380^ protein; M - pre-stained protein weight marker. **b** - Analysis of HPyV12 VP1^364^ production in yeast using Coomassie blue-stained SDS-PAGE. Production of HPyV12 VP1^364^ protein was analyzed in two yeast clones transformed with pFX7-HPyV12-VP1n^364^ plasmid (lines 1–4) and two clones transformed with pFX7-HPyV12-VP1s^364^ plasmid (lines 5–8). In lanes: 1, 3 - whole lysate of both yeast clones transformed with pFX7-HPyV12-VP1n^364^ plasmid; 2, 4 - the soluble fraction recovered after centrifugation of whole lysate of both yeast clones transformed with pFX7-HPyV12-VP1n^364^ plasmid; 5, 7 - whole lysate of both yeast clones transformed with pFX7-HPyV12-VP1s^364^ plasmid; 6, 8 - the soluble fraction recovered after the centrifugation of whole lysate of both yeast clones transformed with pFX7-HPyV12-VP1s^364^; 9 - purified HPyV12-VP1^364^ protein; M - pre-stained protein weight marker (Thermo Fisher Scientific Baltics)

HPyV12 VP1 gene [GenBank:JX308829] encodes a 380-aa-long protein. For production of HPyV12-derived VP1 VLPs in yeast, we used both the native virus gene sequence (HPyV12 VP1n^380^) and a version that was codon-optimized for *S. cerevisiae* expression (HPyV12 VP1s^380^), synthesized by GenScript. Both VP1-encoding genes were cloned into the expression vector, pFX7 [25], and transformed into the yeast strain, AH22-214. Expression of HPyV12 VP1^380^, analyzed using SDS-PAGE, was low using both versions of the gene (Fig. 3a). Our attempts to purify HPyV12 VP1^380^ using sucrose and CsCl gradient centrifugation did not achieve complete purification (Fig. 3a, line 9). Impurities could not be removed with additional centrifugation in sucrose or CsCl, and they remained in the same fractions as the VP1 protein. The yield of partially-purified VP1^380^ was very low: only 0.05 mg per 1 g of wet yeast biomass. Examination of HPyV12 VP1^380^ assembly into VLPs using negative staining electron microscopy demonstrated that some spherical structures could be found, which were approximately 40 nm in diameter and similar to the typical capsomer-structured surface. However, the majority of the recombinant VP1^380^ formed atypical, small (20 nm in diameter) particles or aggregates (Fig. 2j).

**Fig. 4:**
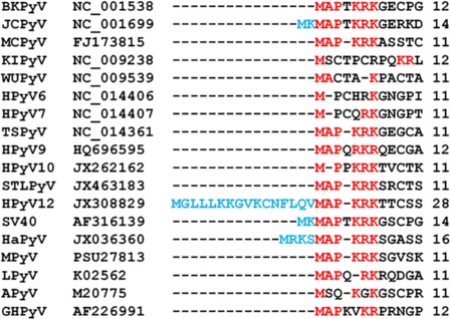
Alignment of VP1 N-terminal aa sequences encoded in the genomes of different PyVs. The aa residues encoded in the first of two potential translation initiation sites are typed in blue; the identical actual N-terminal aa residues are shown in red

In determining why we could not produce HPyV12-derived VP1 VLPs in yeast, we noticed that the N-terminal end of the HPyV12 VP1 protein sequence overhang in the alignment with other VP1 protein sequences derived from different PyVs (Fig. 4). On the other hand, the N-terminal arm encoded within a sequence starting from a second potential translation initiation site in the VP1 gene appeared to be typical of PyV VP1 (Fig. 4). Based on these observations, we decided to express HPyV12 VP1 using a sequence that did not encode an atypical, hydrophobic, 16-aa-long N-terminal peptide. We used the native virus gene sequence (HPyV12 VP1n^364^) and one that was codon-optimized for *S.cerevisiae* expression (HPyV12 VP1s^364^), starting at the second translation initiation site. These were cloned in the vector, pFX7, and transformed into the yeast strain, AH22-214. Production of the 364-aa-long HPyV12 VP1^364^ protein was analyzed using SDS-PAGE. The expression level of VP1^364^ was greatly improved using these sequences compared with using the longer version of the gene (Fig. 3a, b). HPyV12 VP1^364^ was purified using sucrose and CsCl gradient centrifugation. The yields of VP1^364^ were approximately 0.4 mg and 1 mg per 1 g of wet yeast biomass using the native gene and the codon-optimized gene, respectively. The purity and yield of HPyV12 VP1^364^ were similar to those of other HPyV VP1 proteins (Fig. 3b, line 9; Table 1). Thus, using codon-optimized HPyV12 VP1^364^ clearly increased expression and yield of the protein. Analysis of the purified HPyV12 VP1^364^ lacking the 16-aa-long N-terminal using negative staining electron microscopy showed that this protein efficiently assembled into VLPs that were 45–50 nm in diameter (Fig. 2k).

Collectively, these data suggest that the translation initiation site of VP1 was predicted incorrectly by the HPyV12 genome sequence annotated in GenBank. The second translation initiation site in the putative ORF encoding VP1 is more likely the true start site, resulting in efficient translation of a 364-aa-long VP1 protein and it’s self-assembly into VLPs.

### Detection of purified HPyVs derived VP1 proteins by Western blot

**Fig. 5:**
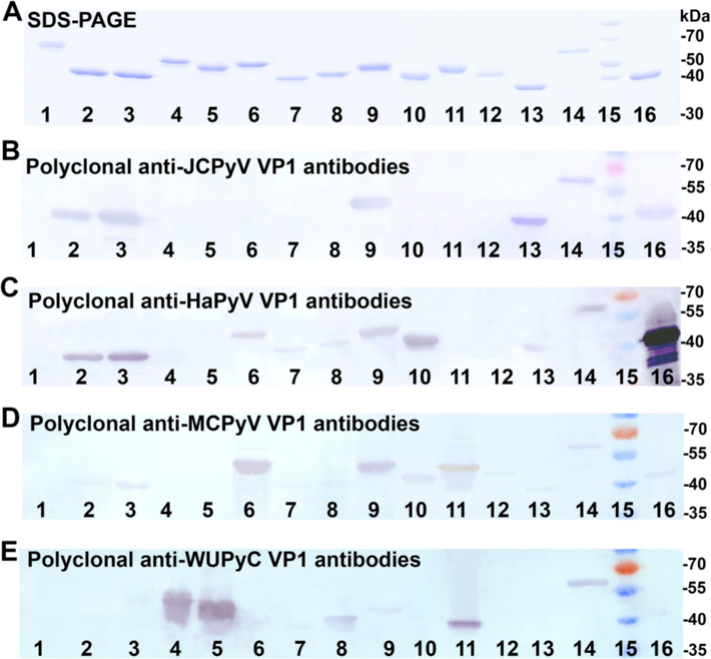
Detection of purified HPyV-derived VP1 VLPs in Western blot with polyclonal antibodies. **a**, Coomassie blue-stained SDS-PAGE; (**b**, **c**, **d**, **e**), Western blot with anti-JCPyV VP1 (**b**), anti-HaPyV VP1 (**c**), anti-MCPyV VP1 (**d**), and anti-WUPyV VP1 (**e**) polyclonal antibodies. The same samples of purified proteins were run on each gel. In lanes: 1 - HPyV16 L1 protein; 2 - BKPyV VP1 protein; 3 - JCPyV VP1 protein; 4 - KIPyV VP1 protein; 5 - WUPyV VP1 protein; 6 - MCPyV VP1 protein; 7 - HPyV6 VP1 protein; 8 - HPyV7 VP1 protein; 9 - TSPyV VP1 protein; 10 - HPyV9 VP1 protein; 11 - HPyV10 VP1 protein; 12 - STLPyV VP1 protein; 13 - HPyV12 VP1 protein; 14 - NJPyV VP1 protein; 15. Protein weight marker (Thermo Fisher Scientific Baltics); 16 - HaPyV VP1 protein

The sequence alignment of VP1 proteins derived from different HPyVs showed 25–78 % degree of identity in amino acid sequence [20], thus it is not likely that all purified VP1 proteins could be recognized by the same antibody in one Western blot. Nevertheless, polyclonal antibodies of some PyVs demonstrated cross-reactivity in Western blots [20, 30]. Therefore, attempts were made to detect the purified VP1 proteins derived from HPyVs based on their cross-reactivities with anti-JCPyV, anti-HaPyV, anti-MCPyV, and anti-WUPyV VP1 polyclonal antibodies. Polyclonal antibody raised against JCPyV-VP1 protein cross-reacted with previously developed BKPyV VP1 protein (78 % identity) [30] as well as with TSPyV (52 % identity), HPyV12 (54 % identity), and HPyV13 (48 identity) VP1 proteins in Western blot (Fig. 5b, lanes 3, 9, 13, 14). A high degree of cross-reactivity of HPyV VP1 proteins was observed with rabbit antiserum raised against HaPyV VP1 protein. This antiserum failed to detect only KIPyV (31 % identity), WUPyV (31 % identity), HPyV10 (47 % identity), and STLPyV (43 % identity) VP1 proteins (Fig. 5c, lanes 4, 5, 11, 12). Interestingly, VP1 proteins derived from HPyV13 and TSPyV were recognized by all four tested polyclonal antibodies despite low similarity of both proteins to WUPyV VP1 (30 and 29 % identity, respectively) (Fig. 5, lines 9 and 14). In contrast, STLPyV VP1 protein was hardly detected by only one antiserum raised against MCPyV VP1 protein (43 % identity) (Fig. 5, line 12). Anti-MCPyV VP1 polyclonal antibody additionally cross-reacted with TSPyV (57 % identity) and HPyV10 (42 % identity) VP1 proteins (Fig. 5d, lines 9 and 11). Anti-WUPyV VP1 polyclonal antibody showed a very strong cross-reactivity with KIPyV VP1 (66 % identity) but also detected HPyV7, HPyV10, and HPyV13 VP1 proteins identical to WUPyV VP1 only by 41, 33, and 48 %, accordingly (Fig. 5e, lines 4, 8, 11, and 14). Thus, the cross-reactivity allowed detection of all purified HPyV VP1 proteins by Western blot using only four polyclonal antibodies.

### HA activity of VP1-derived VLPs from novel PyVs: MCPyV, KIPyV, WUPyV, HPyV6, HPyV7, TSPyV, HPyV9, HPyV10, STLPyV, HPyV12, and NJPyV

**Fig. 6:**
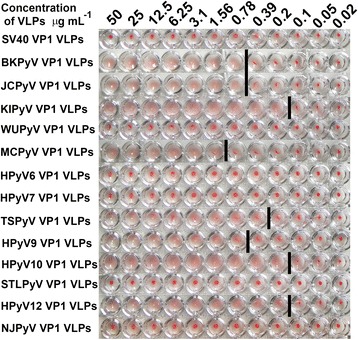
HA activity of HPyV-derived VP1 VLPs. A 2-fold dilution series of purified KIPyV-, WUPyV-, MCPyV-, HPyV6-, HPyV7-, TSPyV-, HPyV9-, HPyV10-, STLPyV-, HPyV12-, and NJPyV-derived VP1 VLPs expressed in *S. cerevisiae* were subjected to a HA assay with 1 % guinea pig erythrocytes. Purified JCPyV- and BKPyV-derived VP1 VLPs were used as positive controls, and SV40-derived VP1 VLPs were used as negative controls. The concentrations of VP1-derived VLPs in μg mL^−1^ are shown on the top. The bar indicates the highest VLP concentration at which HA of guinea pig erythrocytes was observed (HA titer)

All 11 purified VLPs were subjected to HA testing using guinea pig erythrocytes. JCPyV-, BKPyV-, and SV40-derived VP1 VLPs were used as controls. As shown in Fig. 5, the HA titer of purified preparations of JCPyV- and BKPyV-derived VP1 VLPs with known HA activity [37] was down to a concentration of 0.78 μg mL^−1^. JCPyV- and BKPyV-derived VP1 VLPs served as positive controls. The preparation of SV40-derived VP1 VLPs did not show any HA activity using guinea pig erythrocytes and so served as a negative control [37]. HA activity was also confirmed for MCPyV-derived VP1 VLPs with the HA titer down to a concentration of 1.56 μg mL^−1^ [38]. KIPyV-, HPyV10-, and HPyV12-derived VP1 VLPs demonstrated strong HA activity, down to a concentration of 0.2 μg mL^−1^. TSPyV- and HPyV9-derived VP1 VLPs showed HA activity down to a concentration of 0.39 μg mL^−1^ and 0.78 μg mL^−1^, respectively. WUPyV-, HPyV6-, HPyV7-, STLPyV-, and NJPyV-derived VP1 VLPs did not show any HA activity (Fig. 6).

## Discussion

Recently, 11 new HPyVs have been identified. In contrast to the better-studied PyVs, JCPyV, BKPyV, and to a degree, MCPyV, the routes of infection, entry pathways, and cell tropism of these new HPyVs remain unknown. The variety and number of HPyVs indicate that they are capable of self-propagating at low enough levels that do not cause disease, except in immunosuppressed individuals. To advance research on new HPyVs, development of cell culture systems will be important. However, in some applications, VLPs may be used instead, particularly in cases where the wild-type virus is not easily cultivated using cell cultures.

Recombinant VP1 proteins from previously discovered PyVs were successfully produced in *Escherichia coli*, yeast or baculovirus/insect cell expression systems [31]. Yeast or baculovirus/insect cell expression systems were used for efficient production of self-assembled VP1 VLPs derived from BKPyV, JCPyV, HaPyV and other PyVs [29–31]. However, the recombinant VP1 proteins produced in *E. coli* were found mainly as pentamers which self-assembled into VLPs during purification [28, 39]. Only JCPyV- and HaPyV-derived VP1 were shown to form VLPs inside bacteria [40, 41]. Recently large-scale and high-yield production yielding gram-per-liter levels of MPyV VP1 fused with glutathione S-transferase (GST) in *E. coli* was reported [42, 43]. VP1 protein was purified as pentamer and VLPs were reassembled *in vitro* after removal of the GST-tag. It should be noted that the assembly of purified pentamers into VLPs always has a negative impact on the final yield [31], though for MPyV VP1 the VLP reassembly yield was improved and reached 42–56 % [44]. Nevertheless, other authors have shown that yeast-produced MPyV derived VP1 VLPs were more stable as compared to VLPs assembled from pentamers purified from *E. coli* [45]. Similarly, recombinant GST or 6His-tagged VP1 proteins or VP1 pentamers but not VLPs derived from novel HPyVs were produced in bacterial expression systems [15, 46, 47]. To synthesize HPyV-derived VP1 VLPs, mammalian and/or insect cells have been used. MCPyV-derived VP1 VLPs have been generated in mammalian and insect cells [48–50]. HPyV6-, HPyV7-, TSPyV-, HPyV9-, and MWPyV/HPyV10-derived VP1 VLPs have also been successfully produced in insect cells [33, 34, 51]. However, VP1-derived VLPs from STLPyV, HPyV12, and NJPyV have not been generated at all until now.

In this study, we demonstrated that VP1-derived VLPs from 11 recently discovered HPyVs can be efficiently produced in the yeast, *S. cerevisiae*. The synthesis of VLPs using the yeast expression system has several advantages. *S. cerevisiae* is a GRAS (generally recognized as safe) microorganism, free of toxins hazardous to human health [52, 53]. The preparation of products produced in yeast for human use is well established. Additionally, recombinant VP1-derived VLP expression in yeast is relatively cheaper than in insect or mammalian cell cultures, and it results in high yields of VLP [31]. Currently, *S. cerevisiae* is used in the production of vaccines against the recombinant hepatitis B virus (Engerix, Glaxo SmithKline; Gen H-B-Vax, Aventis Pasteur MSD GmbH) and the human papillomavirus (Gardasil^®^) [54].

In this study, to generate 11 new HPyV-derived VP1 proteins in yeast, both native VP1-encoding genes and genes optimized for expression in *S. cerevisiae* were used. The lowest yield (approximately 0.4 mg per 1 g of wet yeast biomass) of purified HPyV10- and HPyV12-derived VP1 protein was obtained using the native gene sequence and NJPyV-derived VP1 protein using gene optimized for expression in *S. cerevisiae* (Table 1). MCPyV-derived VP1 was also expressed using the native gene sequence, but we obtained a high yield of this protein (approximately 0.8 mg per 1 g of wet yeast biomass, Table 1), demonstrating that gene sequence is not the only factor influencing the yield of recombinant protein. The properties of recombinant proteins, stability, resistance to proteases, and solubility, in particular, may have also had an impact on purification efficiency and the final yield. Therefore, the optimization of genes for use in *S. cerevisiae* resulted in only slightly better yields of VP1 from KIPyV, WUPyV, HPyV6, HPyV7, and STLPyV (approximately 0.5–0.6 mg per 1 g of wet yeast biomass) compared to HPyV10-derived VP1. The yield of highly-soluble TSPyV-, HPyV9-, and HPyV12-derived VP1 VLPs, produced using optimized genes, was almost two times greater (approximately 1 mg per 1 g of wet yeast biomass). On the other hand, the difference between the optimized versus native genes was obvious when a shorter HPyV12 VP1 gene version was used. In this case, utilization of optimized VP1 gene improved the yield of VP1s^364^ by a factor of nearly 2.5.

The yeast system supported self-assembly of VP1-derived VLPs from all 11 recently discovered HPyVs. However, HPyV6- and WUPyV-derived VP1 VLP preparations contained mainly smaller-sized capsids. In the case of HPyV6-derived VP1 VLPs (Fig. 2d), small particles were also observed in other hosts. Electron micrographs of HPyV6- and HPyV7-derived VP1 VLPs purified from mammalian 293TT cells show particles smaller than 45–50 nm in diameter [10]. The proportion of small HPyV6-derived VP1 VLPs produced in insect cells was similar to that purified in yeast. Purified HPyV7-derived VP1 VLPs produced both in insect and yeast cells self-assemble into typical PyV-derived VLPs [33]. In contrast, small particles predominate in preparations of HPyV9-dervied VP1 VLPs purified from insect cells [33]. However, in our study, the yeast-generated HPyV9-derived VP1 VLPs assembled into mostly typical PyV-like particles with diameters of approximately 45 nm (Fig. 2g). An explanation for different efficiencies in forming normally-sized VLPs from different PyVs remains elusive. It has been proposed that variation in particle size in SV40 actually indicates the presence of mosaic VLPs, which form from a combination of different geometries of pentamer interactions [55]. Due to numerous possible interactions, SV40 pentamers can self-assemble into a broad array of shapes; this assembly can be redirected by different nucleic acids serving as a scaffold and as a nucleating factor [55]. Similarly, MPyV-derived VP1 self-assembles into VLPs that are 26, 32, and 50 nm in diameter [56]. It has been suggested that the N-terminal arm of VP1 forms a clamp to hold the C-terminal protrusions from neighboring capsomeres in place [57], connecting the N-terminal arms of VP1 from different capsomeres via the intercapsomeric disulphide bridges [45]. Flexibility in this clamping mechanism allows capsomeres to exist in both the pentavalent and hexavalent state [28, 58] and permits the assembly of polymorphic particles in PyVs [56, 59]. On the other hand, sometimes even a single change in an aa residue can influence the size of VP1-derived VLPs. This has been shown in the HaPyV, wherein a single A336G aa exchange in the C-terminal arm of VP1 results in smaller VP1-derived VLPs that are 20 nm in diameter [60]. The HPyV6 and HPyV7 genomes that have been sequenced so far reveal naturally-occurring VP1 aa sequence variation [10]; it is not known how such variation influences VLP assembly. Because smaller particles predominate among HPyV6-derived VP1 VLPs produced in yeast, insect, and mammalian cells, it is possible that some aa variants can support self-assembly of normal-sized HPyV6-derived VP1 VLPs.

As a result of our attempts to express HPyV12-derived VP1 VLPs in yeast and alignment of various PyV-derived VP1 N-terminal sequences, our data suggest that the true translation initiation site predicted by the VP1-encoding ORF is the second translation initiation site [15]. Removal of the 16-aa-long N-terminal peptide greatly improved VP1 expression and VLP formation. There are only a few such cases known in other PyV genomes. HaPyV-, JCPyV-, and SV40-derived VP1 is synthesized starting from the second of two potential translation initiation sites in their respective genes (Fig. 4) [61]. The development of cell culture systems for the production of HPyV12 and determination of the N-terminal sequence of VP1 isolated from the virus capsid could be useful in confirming the true translation initiation start site.

HA activity using guinea pig erythrocytes can expand the number of potential applications for VP1-derived VLPs. They can be used as antigens, not only in enzyme immunoassays, but also in more specific HA inhibition assays to detect corresponding HPyV-specific antibodies in human sera [62]. On the other hand, the HA activity of isolated HPyV-derived VP1 VLPs could serve as an initial index of virus cell-binding properties, which are related to viral spreading, cell tropism, and pathogenicity [63, 64].

The HA activity of JCPyV is associated with the binding of VP1 to sialic acid residues [63–65]. In contrast, SV40 has no HA activity and uses other receptor molecules, e.g., major histocompatibility complex class I molecules [66]. It has been reported that SV40 might bind to sialic acid residues present on ganglioside GM1, but the steric arrangement of the binding amino acids differs from that of hemagglutinating viruses, such as MPyV [67–69]. Consistent with these observations, it has been shown that mutations in the sialic acid binding site impair HA of MCPyV-derived VP1 capsids [70]. The variation in HA activity observed in this study among VP1-derived VLPs from different PyVs might reflect diversity in receptor recognition among PyVs. HA of guinea pig erythrocytes by KIPyV-, TSPyV-, HPyV9-, HPyV10-, and HPyV12-derived VP1 VLPs suggests that these viruses might use sialic acid residues to bind the virus particle to the host cell. In contrast, WUPyV-, HPyV6-, HPyV7-, STLPyV-, and NJPyV-derived VP1 VLPs showed no HA activity and thus more likely use other receptor molecules. Indeed, according to the solved high-resolution crystal structure of HPyV9 VP1 in complex with the three putative oligosaccharide receptors identified by glycan microarray screening, HPyV9 engages short sialylated oligosaccharides [71]. Consistent with these observations, HPyV9-derived VP1 VLPs showed HA of guinea pig erythrocytes in our study. Protein structure analysis of HPyV6- and HPyV7-derived VP1 reveals an obstructed sialic acid binding site [72]. Additionally, VP1 derived from HPyV6 and HPyV7 does not interact with sialylated compounds in solution or in cultured human cells [72]. Stroh et al. suggest that it is still possible that HPyV6 and HPyV7 bind sialic acids at a site in the fully assembled virus, but not in the free VP1 pentamer [72]. In our study, the yeast-produced HPyV6- and HPyV7-derived VP1 VLPs had no HA activity, supporting the recent observations that these two viruses bind non-sialylated receptors. The VP1-derived VLPs from two other members of the genus *Wukipolyomavirus* (KIPyV and WUPyV) differed in their ability to hemagglutinate guinea pig erythrocytes, despite the fact that none of the sialic acid binding residues found in SV40, JCPyV, or MPyV are conserved in the WUPyV and KIPyV VP1 structures [73]. KIPyV-derived VP1 VLPs efficiently hemagglutinated guinea pig erythrocytes, whereas WUPyV-derived VP1 VLPs did not show any HA activity. The data suggest that these two similar viruses might use different receptor molecules to bind host cells.

## Conclusions

The yeast expression system was successfully adapted for high-throughput production of recombinant VP1-derived VLPs from 11 newly identified HPyVs. Although HPyV12 VP1 was predicted to encode a 380-aa-long protein, our findings suggest that translation starts not at the first, but rather at the second, of two potential initiation sites in the VP1-encoding ORF, resulting in a 364-aa-long protein. Yeast-generated recombinant VP1-derived VLPs from different HPyVs demonstrated distinct HA activities and may be useful in virus diagnostics, capsid structure studies, or the investigation of entry pathways and cell tropism of new HPyVs until cell culture systems for new HPyVs are developed.

## Methods

### Generation of yeast expression plasmids harboring VP1 genes

Construction of plasmids and other genetic manipulations were performed according to standard procedures [74] using enzymes and kits purchased from UAB Thermo Fisher Scientific Baltics (Vilnius, Lithuania). Genes encoding VP1 from KIPyV, WUPyV, HPyV6, HPyV7, TSPyV, HPyV9, STLPyV, and NJPyV were codon-optimized for expression in yeast [GenBank KP293742, KP293743, KP293744, KP293745, KP293746, KP293747, and KP293749] and commercially synthesized by GenScript (Piscataway, NJ, USA). The MCPyV VP1-encoding sequence was amplified using Phusion High-Fidelity DNA Polymerase (Thermo Fisher Scientific Baltics) from a healthy volunteer skin swab and then sequenced [GenBank: KP293741]. The samples of human skin swabs were collected and used with the approval of Vilnius Regional Research Ethics Committee (Permit no. 158200-7-070-17) within the project “Development of diagnostic tools for Merkel cell polyomavirus” during 2009–2010. HPyV10 VP1 was synthesized according to the native virus gene sequence without codon optimization, but with two nucleotide changes to remove restriction endonuclease XbaI recognition sites [GenBank: KP293748].

Two versions of the HPyV12 VP1 protein, consisting of a 380-aa chain, were commercially synthesized by GenScript: 1) HPyV12 VP1n^380^ [GenBank: KP293750], according to the native virus gene sequence with one nucleotide changed to remove a restriction endonuclease XbaI recognition site, and 2) HPyV12 VP1s^380^ with a *S. cerevisiae* codon-optimized gene sequence [GenBank: KP293751]. Shorter 364-aa-long versions of the HPyV12 VP1 protein with native (HPyV12 VP1n^364^) and codon-optimized sequences (HPyV12 VP1s^364^) were amplified from HPyV12 VP1n^380^ and HPyV12 VP1s^380^ using Phusion High-Fidelity DNA Polymerase and the primers: HPy12-VP1ATG2-NX: ctctagaatggcacccaagaggaaaaccacctg; HPy12-VP1stop-NX: gtctagattatggaactggtgttatttcttgtc; HPy12-VP1-ATG2-SX: ctctagaatggcacctaaaaggaagaccacatg; HPy12-VP1stopSX: gtctagattatggaactggagtaatttc.

All VP1-encoding DNA fragments were sub-cloned into the XbaI site of the yeast expression vector pFX7 for target protein expression [29]. Recombinants were screened in *Escherichia coli* DH5α cells.

### Yeast cultivation conditions and analysis of VP1 expression

The vector pFX7, without any insert or constructed plasmids, pFX7-KIPyV-VP1, pFX7-WUPyV-VP1, pFX7-MCPyV-VP1, pFX7-HPyV6-VP1, pFX7-HPyV7-VP1, pFX7-TSPyV-VP1, pFX7-HPyV9-VP1, pFX7-HPyV10-VP1, pFX7-STPyV-VP1, pFX7-HPyV12-VP1n^380^, pFX7-HPyV12-VP1s^380^, pFX7-HPyV12-VP1n^364^, pFX7-HPyV12-VP1s^364^, and pFX7-NJPyV-VP1 were transformed into the *S. cerevisiae* strain, AH22-214 (*a, leu2-3,112, his4-519*). The growing conditions for yeast transformants harboring plasmids with VP1-encoding genes from different HPyVs and conditions for induction of VP1 synthesis were similar to that previously described [75]. Briefly, yeast cells were first cultured in glucose- then galactose-containing induction media for 24 and 18 h, respectively. Yeast cells harboring recombinant proteins were collected by centrifugation, washed with distillate water, and then stored at −20 °C until purification or analysis by electrophoresis in 12 % SDS-PAGE. For analysis of VP1 expression, harvested yeast cells (20–50 mg) were suspended in 20–50 μL of DB150 buffer (150 mM NaCl, 1 mM CaCl_2_, 0.25 M L-Arginine, 0.001 % Triton X-100, 10 mM Tris/HCl, pH 7.2), lysed by vortexing with an equal volume of glass beads for 7 min, and then cooled on ice for 1 min between each vortexing step. A 15–30-μL aliquot of each yeast lysate and the supernatant obtained after centrifugation of each yeast lysate were prepared for analysis by mixing with the SDS-PAGE sample buffer (Thermo Fisher Scientific Baltics) and then boiling for 5 min. Prepared samples were then subjected to SDS-PAGE and run in SDS-Tris-glycine buffer. The resultant gel was stained with Coomassie brilliant blue (Sigma-Aldrich, St. Louis, MO, USA) for protein band visualization.

### Purification and characterization of HPyV VP1-derived VLPs

Recombinant VP1 protein purification was carried out as previously described [75]. Briefly, yeast cells containing recombinant VP1 protein were suspended in DB450 buffer (450 mM NaCl, 1 mM CaCl_2_, 0.001 % 0.25 M L-Arginine, and Trition X-100, in 10 mM Tris/HCl-buffer, pH 7.2) with 2 mM PMSF and EDTA-free Complete Protease Inhibitor Cocktail tablets (Roche Diagnostics, Mannheim, Germany), and then were homogenized with glass beads using Bead-Beater GB26 (BioSpec Products, Inc., Bartlesville, USA). The supernatant collected after centrifugation of yeast lysate was loaded onto a 20–69 % sucrose gradient in DB150 buffer and then centrifuged at 100,000 × g (Beckman Optima LE-80 K Ultracentrifuge, Brea, CA, USA) overnight at 4 °C. Thereafter, 2-mL fractions were collected and samples from each fraction were analyzed using Coomassie brilliant blue-stained SDS-PAGE. Fractions containing VP1 were pooled, diluted in DB150 buffer, and after a 0.5 h incubation step with DNAse-free RNAse (Thermo Fisher Scientific Baltics), VP1-derived VLPs were concentrated by ultracentrifugation at 100,000 × g for 4 h at 4 °C. Afterwards, pellets containing VP1 were dissolved in DB150 buffer and subjected to ultracentrifugation on a CsCl gradient with densities ranging from 1.23 to 1.46 g mL^−1^ at 100,000 × g overnight at 4 °C. Fractions were collected and analyzed, as described above. After pooling and diluting purified VP1-containing fractions in DB150 buffer, VP1-derived VLPs were precipitated by ultracentrifugation for 4 h at 100,000 × g. Pellets with VP1-derived VLPs were dissolved in PBS, dialyzed against PBS overnight, aliquoted, and then lyophilized or stored in PBS in 50 % glycerol at −20 °C. JCPyV, BKPyV, SV40, and HaPyV VP1-derived VLPs were prepared as previously described [30].

To confirm VLP assembly of recombinant VP1 proteins generated in yeast, a sample of VP1 purified by ultracentrifugation was placed on 300-mesh carbon-coated palladium grid. VP1-derived VLPs adhering to the grid were negatively stained with 2 % aqueous uranyl acetate solution and examined with a Morgagni 268 electron microscope (FEI Inc., Hillsboro, OR, USA).

### Western blot

Samples of purified recombinant VP1 proteins derived from all HPyVs and HaPyV were mixed with the SDS-PAGE sample buffer (Thermo Fisher Scientific Baltics), boiled for 5 min, applied to a SDS-PAGE and run in SDS-Tris-glycine buffer. Protein bands in SDS-PAGE were electro-transferred to Immobilon P membrane (Millipore, Bedford, MA, USA) or visualized by staining with Coomassie brilliant blue (Sigma-Aldrich, St. Louis, MO, USA). The membranes were blocked with 5 % milk in TTBS (0.1 % Tween 20, 0.1 M Tris, 0.3 M NaCl, pH 7.4) and incubated overnight in the relevant antibody solutions at room temperature. Anti-JCPyV, −MCPyV, and -WUPyV VP1 mouse sera (raised against the respective VP1 protein) and anti-HaPyV VP1 rabbit serum [75] were diluted 1:500, 1:300, 1:300, and 1:1000, respectively, in TTBS. After incubation with the diluted antibodies, the membranes were incubated for 2 h with horseradish peroxidase-labeled anti-mouse or anti-rabbit IgG conjugate (Bio-Rad, Hercules, CA, USA) diluted 1:1000. The peroxidase-mediated protein band staining was performed by adding 4-chloro-1-naphtol (BioChemica, Darmstadt, Germany) and H_2_O_2_ (Roth, Karlsruhe, Germany).

### Hemagglutination assay

VLPs were subjected to a HA assay using guinea pig erythrocytes in a U-shaped microtiter plate. VLPs of each purified VP1 protein were serially diluted (50 μg mL^−1^ to 0.025 μg mL^−1^) in 25 μL PBS and mixed with 25 μL of a 1 % (v/v) guinea pig erythrocyte suspension in PBS. HA activity was recorded after a 3 h incubation at 4 °C. The HA titer was defined as the reciprocal of the highest antigen dilution clearly showing HA.

## Abbreviations

AIDS: Acquired immune deficiency syndrome
BKPyV: BK polyomavirus
HA: Hemagglutination
HaPyV: Hamster polyomavirus
HPyVs: Human polyomaviruses
GASH:Sal: Genetic audiogenic seizure hamsters
GST: Glutathione S-transferase
GRAS: Generally recognized as safe
JCPyV: JC polyomavirus
KIPyV: Karolinska Institutet polyomavirus
MCPyV: Merkel cell polyomavirus
MPyV: Murine polyomavirus
MWPyV: Malawi polyomavirus
MXPyV: Mexico polyomavirus
NJPyV: New Jersey polyomavirus
ORF: Open reading frame
PCR: Polymerase-chain reaction
PyVs: Polyomaviruses
SDS-PAGE: Sodium dodecyl sulfate-polyacrylamide gels
STLPyV: St. Louis polyomavirus
SV40: Simian virus 40
TSPyV: Trichodysplasia spinulosa-associated polyomavirus
VLPs: Virus-like particles
WUPyV: Washington University polyomavirus

## References

[1] Imperiale MJ, Major EO, Knipe DM, Howley PM, Griffin DE, Lamb RA, Martin MA, Roizman B, Straus SE (2007). Polyomaviruses. Fields virology.

[2] Feltkamp MC, Kazem S, van der Meijden E, Lauber C, Gorbalenya AE (2013). From Stockholm to Malawi: recent developments in studying human polyomaviruses. J Gen Virol.

[3] Johne R, Buck CB, Allander T, Atwood WJ, Garcea RL, Imperiale MJ (2011). Taxonomical developments in the family Polyomaviridae. Arch Virol.

[4] Stewart SE, Eddy BE, Borgese NG (1958). Neoplasms in mice inoculated with a tumor agent carried in tissue culture. J Natl Cancer Inst.

[5] Gardner SD, Field AM, Coleman DV, Hulme B (1971). New human papovavirus (B.K.) isolated from urine after renal transplantation. Lancet.

[6] Padgett BL, Walker DL, ZuRhein GM, Eckroade RJ, Dessel BH (1971). Cultivation of papova-like virus from human brain with progressive multifocal leucoencephalopathy. Lancet.

[7] Allander T, Andreasson K, Gupta S, Bjerkner A, Bogdanovic G, Persson MA (2007). Identification of a third human polyomavirus. J Virol.

[8] Gaynor AM, Nissen MD, Whiley DM, Mackay IM, Lambert SB, Wu G (2007). Identification of a novel polyomavirus from patients with acute respiratory tract infections. PLoS Pathog.

[9] Feng H, Shuda M, Chang Y, Moore PS (2008). Clonal integration of a polyomavirus in human Merkel cell carcinoma. Science.

[10] Schowalter RM, Pastrana DV, Pumphrey KA, Moyer AL, Buck CB (2010). Merkel cell polyomavirus and two previously unknown polyomaviruses are chronically shed from human skin. Cell Host Microbe.

[11] van der Meijden E, Janssens RW, Lauber C, Bouwes Bavinck JN, Gorbalenya AE, Feltkamp MC (2010). Discovery of a new human polyomavirus associated with trichodysplasia spinulosa in an immunocompromized patient. PLoS Pathog.

[12] Scuda N, Hofmann J, Calvignac-Spencer S, Ruprecht K, Liman P, Kühn J (2011). A novel human polyomavirus closely related to the african green monkey-derived lymphotropic polyomavirus. J Virol.

[13] Buck CB, Phan GQ, Raiji MT, Murphy PM, McDermott DH, McBride AA (2012). Complete genome sequence of a tenth human polyomavirus. J Virol.

[14] Lim ES, Reyes A, Antonio M, Saha D, Ikumapayi UN, Adeyemi M (2013). Discovery of STL polyomavirus, a polyomavirus of ancestral recombinant origin that encodes a unique T antigen by alternative splicing. Virology.

[15] Korup S, Rietscher J, Calvignac-Spencer S, Trusch F, Hofmann J, Moens U (2013). Identification of a novel human polyomavirus in organs of the gastrointestinal tract. PLoS One.

[16] Siebrasse EA, Reyes A, Lim ES, Zhao G, Mkakosya RS, Manary MJ (2012). Identification of MW polyomavirus, a novel polyomavirus in human stool. J Virol.

[17] Yu G, Greninger AL, Isa P, Phan TG, Martinez MA, de la Luz Sanchez M (2012). Discovery of a novel polyomavirus in acute diarrheal samples from children. PLoS One.

[18] Mishra N, Pereira M, Rhodes RH, An P, Pipas J, Jain K (2014). Identification of a novel polyomavirus in a pancreatic transplant recipient with retinal blindness and vasculitic myopathy. J Infect Dis.

[19] Dalianis T, Hirsch HH (2013). Human polyomaviruses in disease and cancer. Virology.

[20] Moens U, Van Ghelue M, Song X, Ehlers B (2013). Serological cross-reactivity between human polyomaviruses. Rev Med Virol.

[21] Knowles WA, Khalili K, Stoner GL (2001). Serendipity – the discovery of BK virus. Human polyomaviruses: molecular and clinical perspectives.

[22] Pinto M, Dobson S (2014). BK and JC virus: a review. J Infect.

[23] Ferenczy MW, Marshall LJ, Nelson CD, Atwood WJ, Nath A, Khalili K (2012). Molecular biology, epidemiology, and pathogenesis of progressive multifocal leukoencephalopathy, the JC virus-induced demyelinating disease of the human brain. Clin Microbiol Rev.

[24] Ho J, Jedrych JJ, Feng H, Natalie AA, Grandinetti L, Mirvish E, et al. Human polyomavirus 7-associated pruritic rash and viremia in transplant recipients. J Infect Dis. 2014. doi: 10.1093/infdis/jiu524. 10.1093/infdis/jiu524 PMC4425822 25231015

[25] Munoz LJ, Ludena D, Gedvilaite A, Zvirbliene A, Jandrig B, Voronkova T (2013). Lymphoma outbreak in a GASH:Sal hamster colony. Arch Virol.

[26] Cheng J, DeCaprio JA, Fluck MM, Schaffhausen BS (2009). Cellular transformation by Simian Virus 40 and Murine Polyoma Virus Tantigens. Semin Cancer Biol.

[27] Shen PS, Enderlein D, Nelson CD, Carter WS, Kawano M, Xing L, Swenson RD (2011). The structure of avian polyomavirus reveals variably sized capsids, nonconserved inter-capsomere interactions, and a possible location of the minor capsid protein VP4. Virology.

[28] Salunke DM, Caspar DL, Garcea RL (1986). Self-assembly of purified polyomavirus capsid protein VP1. Cell.

[29] Sasnauskas K, Buzaite O, Vogel F, Jandrig B, Razanskas R, Staniulis J (1999). Yeast cells allow the high-level expression and formation of polyomavirus-like particles. Biol Chem.

[30] Sasnauskas K, Bulavaite A, Hale A, Jin L, Gedvilaite A, Dargeviciute A (2002). Generation of recombinant virus-like particles of human and non-human polyomaviruses in yeast *Saccharomyces cerevisiae*. Intervirology.

[31] Teunissen EA, de Raad M, Mastrobattista E (2013). Production and biomedical applications of virus-like particles derived from polyomaviruses. J Control Release.

[32] Nelson CD, Derdowski A, Maginnis MS, O’Hara BA, Atwood WJ (2012). The VP1 subunit of JC polyomavirus recapitulates early events in viral trafficking and is a novel tool to study polyomavirus entry. Virology.

[33] Nicol JT, Robinot R, Carpentier A, Carandina G, Mazzoni E, Tognon M (2013). Age-specific seroprevalences of merkel cell polyomavirus, human polyomaviruses 6, 7, and 9, and trichodysplasia spinulosa-associated polyomavirus. Clin Vaccine Immunol.

[34] Nicol JT, Leblond V, Arnold F, Guerra G, Mazzoni E, Tognon M (2014). Seroprevalence of Human Malawi Polyomavirus. J Clin Microbiol.

[35] Norkiene M, Gedvilaite A (2012). Influence of codon bias on heterologous production of human papillomavirus type 16 major structural protein L1 in yeast. Sci World J.

[36] Angov E, Hillier CJ, Kincaid RL, Lyon JA (2008). Heterologous protein expression is enhanced by harmonizing the codon usage frequencies of the target gene with those of the expression host. PLoS One.

[37] Rodgers RE, Chang D, Cai X, Consigli RA (1994). Purification of recombinant budgerigar fledgling disease virus VP1 capsid protein and its ability for in vitro capsid assembly. J Virol.

[38] Knowles WA, Pipkin P, Andrews N, Vyse A, Minor P, Brown DW (2003). Population-based study of antibody to the human polyomaviruses BKVand JCV and the simian polyomavirus SV40. J Med Virol.

[39] Erickson KD, Garcea RL, Tsai B (2009). Ganglioside GT1b is a putative host cell receptor for the Merkel cell polyomavirus. J Virol.

[40] Ou WC, Wang M, Fung CY, Tsai RT, Chao PC, Hseu TH (1999). The major capsid protein, VP1, of human JC virus expressed in Escherichia coli is able to self-assemble into a capsid-like particle and deliver exogenous DNA into human kidney cells. J Gen Virol.

[41] Voronkova T, Kazaks A, Ose V, Ozel M, Scherneck S, Pumpens P (2007). Hamster polyomavirus-derived virus-like particles are able to transfer in vitro encapsidated plasmid DNA to mammalian cells. Virus Genes.

[42] Liew MW, Rajendran A, Middelberg AP (2010). Microbial production of virus-like particle vaccine protein at gram-per-litre levels. J Biotechnol.

[43] Middelberg AP, Rivera-Hernandez T, Wibowo N, Lua LH, Fan Y, Magor G (2011). A microbial platform for rapid and low-cost virus-like particle and capsomere vaccines. Vaccine.

[44] Liew MW, Chuan YP, Middelberg AP (2012). High-yield and scalable cell-free assembly of virus-like particles by dilution. Biochem Eng J.

[45] Simon C, Klose T, Herbst S, Han BG, Sinz A, Glaeser RM (2014). Disulfide linkage and structure of highly stable yeast-derived virus-like particles of murine polyomavirus. J Biol Chem.

[46] Kean JM, Rao S, Wang M, Garcea RL (2009). Seroepidemiology of human polyomaviruses. PLoS Pathog.

[47] van der Meijden E, Kazem S, Burgers MM, Janssens R, Bouwes Bavinck JN, de Melker H (2011). Seroprevalence of Trichodysplasia Spinulosa–associated Polyomavirus. Emerg Infect Dis.

[48] Pastrana DV, Tolstov YL, Becker JC, Moore PS, Chang Y, Buck CB (2009). Quantitation of human seroresponsiveness to Merkel cell polyomavirus. PLoS Pathog.

[49] Tolstov YL, Pastrana DV, Feng H, Becker JC, Jenkins FJ, Moschos S (2009). Human Merkel cell polyomavirus infection II. MCV is a common human infection that can be detected by conformational capsid epitope immunoassays. Int J Cancer.

[50] Touze A, Gaitan J, Arnold F, Cazal R, Fleury MJ, Combelas N (2010). Generation of Merkel cell polyomavirus (MCV)-like particles and their application to detection of MCV antibodies. J Clin Microbiol.

[51] Kumar A, Kantele A, Jarvinen T, Chen T, Kavola H, Sadeghi M (2012). Trichodysplasia spinulosa-associated polyomavirus (TSV) and Merkel cell polyomavirus: correlation between humoral and cellular immunity stronger with TSV. PLoS One.

[52] Canganella F, Paganini S, Ovidi M, Vettraino AM, Bevilacqua L, Massa S (1997). A microbiology investigation on probiotic pharmaceutical products used for human health. Microbiol Res.

[53] Garrait G, Jarrige JF, Blanquet S, Beyssac E, Alric M (2007). Recombinant *Saccharomyces cerevisiae* strain expressing a model cytochrome P450 in the rat digestive environment: viability and bioconversion activity. Appl Environ Microbiol.

[54] Villa LL, Costa RL, Petta CA, Andrade RP, Ault KA, Giuliano AR (2005). Prophylactic quadrivalent human papillomavirus (types 6, 11, 16, and 18) L1 virus-like particle vaccine in young women: a randomised double-blind placebo-controlled multicentre phase II efficacy trial. Lancet Oncol.

[55] Kler S, Wang JC, Dhason M, Oppenheim A, Zlotnick A (2013). Scaffold properties are a key determinant of the size and shape of self-assembled virus-derived particles. ACS Chem Biol.

[56] Salunke DM, Caspar DL, Garcea RL (1989). Polymorphism in the assembly of polyomavirus capsid protein VP1. Biophys J.

[57] Stehle T, Harrison S (1996). Crystal structures of murine polyomavirus in complex with straight-chain and branched-chain sialyloligosaccharide receptor fragment. Structure.

[58] Rayment I, Baker TS, Caspar DL, Murakami WT (1982). Polyoma virus capsid structure at 22.5 A resolution. Nature.

[59] Nilsson J, Miyazaki N, Xing L, Wu B, Hammar L, Li TC (2005). Structure and assembly of a T = 1 virus-like particle in BK polyomavirus. J Virol.

[60] Gedvilaite A, Aleksaite E, Staniulis J, Ulrich R, Sasnauskas K (2006). Size and position of truncations at the carboxy-terminal region of major capsid protein VP1 of hamster polyomavirus expressed in yeast determine its assembly capacity. Arch Virology.

[61] Siray H, Ozel M, Jandrig B, Voronkova T, Jia W, Zocher R (1999). Capsid protein-encoding genes of hamster polyomavirus and properties of the viral capsid. Virus Genes.

[62] Knowles WA, Sasnauskas K (2003). Comparison of cell culture-grown JC virus (primary human fetal glial cells and the JCI cell line) and recombinant JCV VP1 as antigen for the detection of anti-JCV antibody by haemagglutination inhibition. J Virol Methods.

[63] O’Hara SD, Stehle T, Garcea R (2014). Glycan receptors of the Polyomaviridae: structure, function, and pathogenesis. Curr Opin Virol.

[64] Maginnis MS, Nelson CD, Atwood WJ. JC polyomavirus attachment, entry, and trafficking: unlocking the keys to a fatal infection. J Neurovirol. 2014. [Epub ahead of print]. 10.1007/s13365-014-0272-4 PMC4312552 25078361

[65] Stehle T, Yan Y, Benjamin TL, Harrison SC (1994). Structure of murine polyomavirus complexed with an oligosaccharide receptor fragment. Nature.

[66] Breau WC, Atwood WJ, Norkin LC (1992). Class I major histocompatibility proteins are an essential component of the simian virus 40 receptor. J Virol.

[67] Neu U, Woellner K, Gauglitz G, Stehle T (2008). Structural basis of GM1 ganglioside recognition by simian virus 40. Proc Natl Acad Sci U S A.

[68] Ewers H, Romer W, Smith AE, Bacia K, Dmitrieff S, Chai W (2010). GM1 structure determines SV40-induced membrane invagination and infection. Nat Cell Biol.

[69] Magaldi TG, Buch MH, Murata H, Erickson KD, Neu U, Garcea RL (2012). Mutations in the GM1 binding site of simian virus 40 VP1 alter receptor usage and cell tropism. J Virol.

[70] Neu U, Hengel H, Blaum BS, Schowalter RM, Macejak D, Gilbert M (2012). Structures of Merkel cell polyomavirus VP1 complexes define a sialic acid binding site required for infection. PLoS Pathog.

[71] Khan ZM, Liu Y, Neu U, Gilbert M, Ehlers B, Feizi T (2014). Crystallographic and glycan microarray analysis of human polyomavirus 9 VP1 identifies N-glycolyl neuraminic acid as a receptor candidate. J Virol.

[72] Stroh LJ, Neu U, Blaum BS, Buch MH, Garcea RL, Stehle T (2014). Structure analysis of the major capsid proteins of human polyomaviruses 6 and 7 reveals an obstructed sialic acid binding site. J Virol.

[73] Neu U, Wang J, Macejak D, Garcea RL, Stehle T (2011). Structures of the major capsid proteins of the human Karolinska Institutet and Washington University polyomaviruses. J Virol.

[74] Sambrook J, Russell DW (2001). Molecular cloning, a laboratory manual.

[75] Pleckaityte M, Zvirbliene A, Sezaite I, Gedvilaite A (2011). Production in yeast of pseudotype virus-like particles harboring functionally active antibody fragments neutralizing the cytolytic activity of vaginolysin. Microb Cell Fact.

